# Applying the *M*_*2*_ Statistic to Evaluate the Fit of Diagnostic Classification Models in the Presence of Attribute Hierarchies

**DOI:** 10.3389/fpsyg.2018.01875

**Published:** 2018-10-09

**Authors:** Fu Chen, Yanlou Liu, Tao Xin, Ying Cui

**Affiliations:** ^1^Faculty of Psychology, Beijing Normal University, Beijing, China; ^2^China Academy of Big Data for Education, Qufu Normal University, Shandong, China; ^3^Collaborative Innovation Center of Assessment toward Basic Education Quality, Beijing Normal University, Beijing, China; ^4^Department of Educational Psychology, University of Alberta, Edmonton, AB, Canada

**Keywords:** diagnostic classification models, attribute hierarchies, absolute fit test, limited-information test statistics, goodness-of-fit

## Abstract

The performance of the limited-information statistic *M*_*2*_ for diagnostic classification models (DCMs) is under-investigated in the current literature. Specifically, the investigations of *M*_*2*_ for specific DCMs rather than general modeling frameworks are needed. This article aims to demonstrate the usefulness of *M*_*2*_ in hierarchical diagnostic classification models (HDCMs). The performance of *M*_*2*_ in evaluating the fit of HDCMs was investigated in the presence of four types of attribute hierarchies. Two simulation studies were conducted to examine Type I error rates and statistical power of *M*_*2*_ under different simulation conditions, respectively. The findings suggest acceptable Type I error rates control of *M*_*2*_ as well as high statistical power under the conditions of a Q-matrix misspecification and the DINA model misspecification. The data of Examination for the Certificate of Proficiency in English (ECPE) were used to empirically illustrate the suitability of *M*_*2*_ in practice.

## Introduction

Diagnostic classification models (DCMs) (Rupp et al., 2010) have demonstrated great potential for evaluating respondents with fine-grained information to support targeted interventions. Previous studies have applied DCMs to address some practical issues in education (e.g., Jang, 2009) and psychology (e.g., Templin and Henson, 2006). However, the area of research in DCMs is still relatively new. More research on model data fit statistics are needed for evaluating DCMs. Although relative fit statistics (e.g., de la Torre and Douglas, 2008) are available for determining the most suitable of several alternative models, they cannot be used for evaluating test- or item-level goodness-of-fit. As such, some authors have proposed and developed methods to evaluate the absolute fit of DCMs (e.g., Jurich, 2014; Wang et al., 2015). Specifically, the limited-information test statistics, e.g., the *M*_*2*_ statistic, was recommended by researchers (e.g., Liu et al., 2016) because of its ability to address sparseness in the contingency table. In the study by Liu et al. (2016), the performance of *M*_*2*_ was evaluated under the log-linear cognitive diagnosis model (LCDM; Henson et al., 2009). They found that *M*_*2*_ has reasonable Type I error rates control when models were correctly specified and good statistical power when models or Q-matrix were misspecified, under the conditions of different sample sizes, test lengths, and attribute correlations. More importantly, their study has identified the cutoff values of the root mean square error of approximation (RMSEA) fit index for *M*_*2*_ (RMSEA_*2*_). Similarly, the study by Henson et al. (2009) validated the usefulness of *M*_*2*_ in DCMs by a series simulation studies. Specifically, their study showed that *M*_*2*_ is of good performance across different diagnostic model structures and is sensitive to the model misspecifications, the Q-matrix misspecifications, the misspecifications in the distribution of higher-order latent dimensions, and violations of local item independence. Generally, their findings were based on the general frameworks (e.g., LCDM) or the most common DCMs (e.g., DINA; de la Torre, 2009), which assume extremely complicated relationships among items but simple relationships among attributes. However, the more specific models are more suitable for practical use (Rojas et al., 2012; Ma et al., 2016), the results will be more convincing if *M*_*2*_ can be applied in more specific and practical conditions.

In education, students are often required to master certain requisite skills before they move on to learn new knowledge and skills. This indicates that hierarchical relationships often exist among the cognitive skills. To address the presence of hierarchical relationship among attributes, Templin and Bradshaw (2014) developed the hierarchical diagnostic classification models (HDCMs) to model the attribute hierarchies. Hence, applying *M*_*2*_ to evaluate the fit of the DCMs in the presence of attribute hierarchies can help further testify to the utility of limited-information tests for DCMs. In this study, we use *M*_*2*_ and examine its Type I error rates and its power to evaluate the overall fit of HDCMs under different simulation conditions. Specifically, five types of DCMs are considered in our research: LCDM and HDCMs with linear, convergent, divergent and unstructured attribute hierarchies. In addition, the performance of *M*_*2*_ is examined with real data.

## Hierarchical diagnostic classification models

Over the past several decades, numerous DCMs have been developed and presented in the psychometric literature. Some of these models are general modeling frameworks, under which other specific DCMs can be subsumed through statistical constraints on model parameters. Hence, the general DCMs can flexibly model the probabilities of examinee's differently structured responses at the sacrifice of model simplicity. In this study, we use as the fitting model the HDCM, which is developed based upon the LCDM framework (Henson et al., 2009). The LCDM defines the probability of a correct response of the *i*th examinee for item *j* as

(1) $$P(X_{ij}=1|\alpha_{i})=\frac{\exp(\lambda_{j,0}+{\lambda^{\prime }}_{j}\text{h}(\alpha_{i},q_{j}))}{1+\exp(\lambda_{j,0}+{\lambda^{\prime }}_{j}h(\alpha_{i},q_{j}))},$$

where $\alpha_{i}={\left(\alpha_{i1},\ldots ,\alpha_{iK}\right)}^{\prime }$ represents the attribute mastery pattern of examinee *i*, λ_*j*, 0_ represents the intercept parameter for item *j*, and $\lambda_{j}^{}$ represents the main and the interaction effect parameters for item *j*. ${\text{q}}_{j}={\left(q_{j1},\ldots ,q_{jk},\ldots ,q_{jK}\right)}^{\prime }$is the Q-matrix entries for the item *j*, where *q*_*jk*_ denotes whether attribute *k* is required by item *j*. ***h*** is a mapping function and is used to indicate the linear combination of ***α***_*i*_ and ***q***_*j*_:

(2) $$\begin{array}{l} \lambda {\;}^{\prime }{}_{j}h(\alpha_{i},q_{j})=\sum_{k=1}^{K}\lambda_{j,1,(k)}\alpha_{ik}q_{jk}\\ \;+\sum_{k=1}^{K-1}\sum_{k^{\prime }>k}\lambda_{j,2,(k,k^{\prime })}\alpha_{ik}\alpha_{ik^{\prime }}q_{jk}q_{jk^{\prime }}+\cdots . \end{array}$$

In the above equation, for item *j*, all main and interaction effects are included. Specifically, λ_*j*,1, (*k*)_ refers to the main effect of attribute *k* for item *j*, and $\lambda_{j,2,\left(k,k^{\prime }\right)}$ refers to the two-way interaction effect of attributes *k* and *k*′ for item *j*. Hence, the subscript following the first comma in λ_*j*, 1, (*k*)_ or $\lambda_{j,2,\left(k,k^{\prime }\right)}$ indicates the effect level, and the subscript(s) in parentheses refers to the involved attribute(s) for the effect. For example, if ${\text{q}}_{j}={\left(0,1,1\right)}^{\prime }$ and $\alpha_{i}={\left(1,1,0\right)}^{\prime }$, namely the second and third attributes, are required by item *j* and the examinee has mastered the first two attributes, then ${\lambda {\;}^{\prime }}_{j}\text{h}\left(\alpha_{i},{\text{q}}_{j}\right)=\lambda_{j,1,\left(2\right)}$. For $\alpha_{i}={\left(0,1,1\right)}^{\prime }$, namely the examinee has mastered all attributes required by item *j*, then $\lambda_{j}^{\text{T}}h\left(\alpha_{i},{\text{q}}_{j}\right)=\lambda_{j,1,\left(2\right)}+\lambda_{j,1,\left(3\right)}+\lambda_{j,2,\left(2,3\right)}$, indicating that two main effects and one two-way interaction effect of attributes 2 and 3 are included in item *j*. Further, a statistical constraint is defined to ensure the monotonicity of the LCDM (see Henson et al., 2009).

Attribute hierarchies representing student knowledge structures often exist in education. However, how to model the attribute hierarchies had not been resolved until the HDCMs were developed by Templin and Bradshaw (2014). As previously mentioned, the LCDM is a general modeling framework under which other specific models can be obtained through statistical constraints. The parameterization of the HDCM, which is based on the LCDM, is no exception. Moreover, because the different types of attribute hierarches correspond to different parameterizations, the linear hierarchies are used for illustration.

With respect to the LCDM, *K* attributes correspond to 2^*K*^ attribute mastery patterns. However, for a linear hierarchy with *K* attributes, the number of attribute mastery patterns is sharply reduced from 2^*K*^ to*K* + 1. This change is reflected by $\alpha_{i}^{*}$, which refers to the possible attribute mastery patterns under the constraints of the linear hierarchy. Allowing ${\text{q}}_{j}={\left(a,b,c,\ldots \right)}^{\prime }$ to denote the attributes required by item *j*, the linear hierarchy defines attribute *a* to be the most fundamental attribute such that any other one attribute is nested within its former attribute. Thus, the matrix product in Equation (2) is modified to ${\lambda {\;}^{\prime }}_{j}\text{h}\left(\alpha_{i}^{*},{\text{q}}_{j}\right)$:

(3) $$\begin{array}{l} \lambda {\;}^{\prime }{}_{j}h(\alpha_{i}^{*},q_{j})=\lambda_{j,1,(a)}\alpha_{ia}q_{ja}+\lambda_{j,2,(b(a))}\alpha_{ia}\alpha_{ib}q_{ja}q_{jb}+\\ \;\lambda_{j,3,(c(b,a))}\alpha_{ia}\alpha_{ib}\alpha_{ic}q_{ja}q_{jb}q_{jc}+\cdots . \end{array}$$

As such, if item *j* measures attributes 1 and 2 and attribute 2 is nested within attribute 1, the HDCM defines the probability of a correct response of the *i*th examinee for item *j* as

(4) $$\begin{array}{l} P(X_{ij}=1|\alpha_{i}^{*})\\ \;=\frac{\exp(\lambda_{j,0}+\lambda_{j,1,(1)}\alpha_{i1}q_{j1}+\lambda_{j,2,(2(1))}\alpha_{i1}\alpha_{i2}q_{j1}q_{j2})}{1+\exp(\lambda_{j,0}+\lambda_{j,1,(1)}\alpha_{i1}q_{j1}+\lambda_{j,2,(2(1))}\alpha_{i1}\alpha_{i2}q_{j1}q_{j2})}. \end{array}$$

Under the HDCM, the item parameters include only one intercept, one main effect for attribute 1 and one interaction effect for attribute 2 nested within attribute 1. Obviously, regardless of the number of attributes, there will be only one main effect in the item response function owing to the constraints of the linear hierarchies; accordingly, the number of item parameters for items measuring several attributes is also greatly reduced.

**Figure 1:**
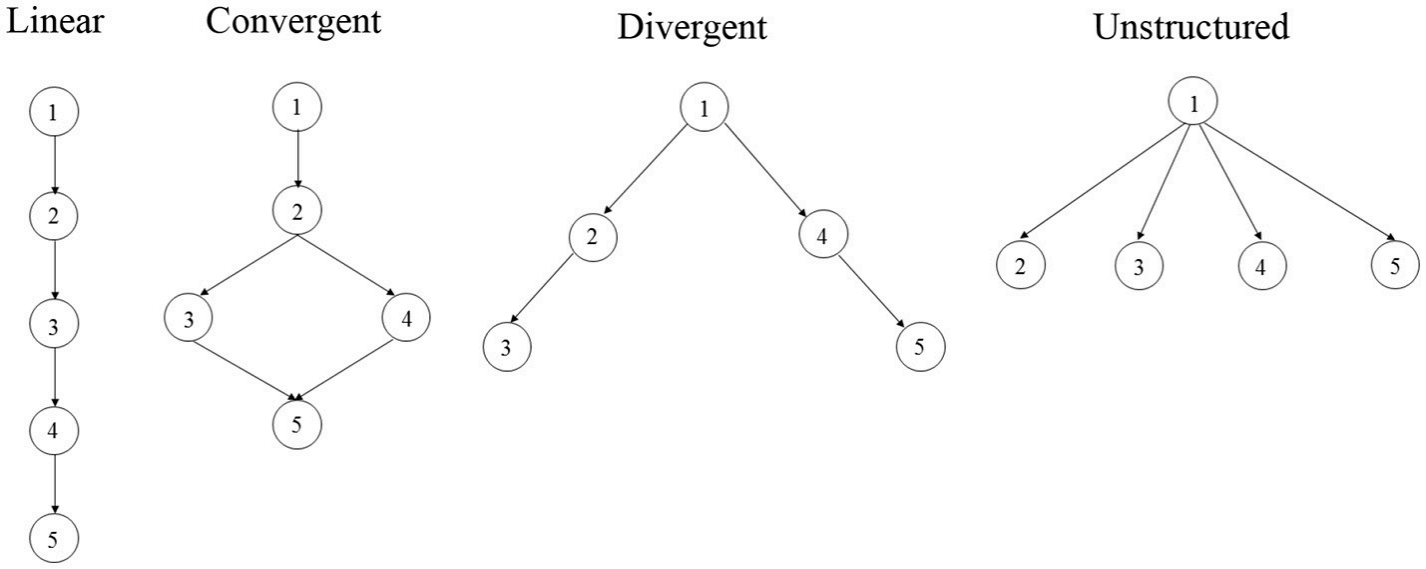
Four hierarchical structures using five attributes.

In this study, four fundamental hierarchical structures (see Figure 1) by Leighton et al. (2004) were modeled by HDCMs. All attribute hierarchies involve five attributes. Specifically, linear hierarchy defines linear relationships between attributes: the mastery of an attribute is dependent on the mastery of its former attribute. As such, attribute 1 is the most fundamental attribute given that it is required for the mastery of any other attributes in the hierarchical structure. In other words, for example, it is unlikely that an examinee have mastery of attributes 3 and 4 without the mastery of attributes 1 and 2. The linear hierarchy would largely reduce the item parameters of LCDM. As an example, assume item *j* measures attributes 1, 2, and 4, the linear HDCM defines the matrix product of item *j* as

(5) $$\begin{array}{l} \lambda_{j,1,(1)}\alpha_{i1}q_{j1}+\lambda_{j,2,(2(1))}\alpha_{i1}\alpha_{i2}q_{j1}q_{j2}\\ \;+\lambda_{j,3,(1,2,4)}\alpha_{i1}\alpha_{i2}\alpha_{i4}q_{j1}q_{j2}q_{j4}. \end{array}$$

It can be found that only the main effect of attribute 1, the two-way interaction effect of attributes 1 and 2, and the three-way interaction effect of attributes 1, 2, and 4 are modeled in HDCM under the constraints of the linear hierarchy. In the convergent hierarchy, the mastery of attributes 3 or 4 are dependent on the mastery of attributes 1 and 2, and both attributes 3 and 4 are prerequisite attributes of attribute 5. As such, the same item measuring attributes 1, 2, and 4 is modeled by the same way as equation (5) under the convergent HDCM. In the divergent hierarchy, attribute 1 is the prerequisite attribute of both attributes 2 and 4, which in turn are the prerequisite attributes of attributes 3 and 5 respectively. Under the divergent HDCM, the same item would be modeled as

(6) $$\begin{array}{l} \lambda_{j,1,(1)}\alpha_{i1}q_{j1}+\lambda_{j,2,(2(1))}\alpha_{i1}\alpha_{i2}q_{j1}q_{j2}+\lambda_{j,2,(4(1))}\alpha_{i1}\alpha_{i4}q_{j1}q_{j4}\\ \;+\lambda_{j,3,(1,2,4)}\alpha_{i1}\alpha_{i2}\alpha_{i4}q_{j1}q_{j2}q_{j4}. \end{array}$$

In the unstructured hierarchy, attribute 1 is the prerequisite attribute of all other attributes, which are independent from each other. Under the unstructured HDCM, the same item would be modeled by the same way of divergent HDCM. It should be noted that for items measuring four or five attributes, different attribute hierarchies would model the same item in more distinct ways, since more attributes lead to more main and interaction effects.

## The *M*_*2*_ test statistic

Regarding the issue of fit tests for DCMs, several recent studies have focused on item-level fit statistics for DCMs (e.g., Kunina-Habenicht et al., 2012; Wang et al., 2015). Despite the feasibility of the proposed item-level fit statistics, a way to assess the absolute model-data fit in the test level remains undeveloped because the traditional full-information statistics, such as χ^2^ and *G*^2^, cannot be practically feasible in DCMs. Specifically, the computations of χ^2^ and *G*^2^ should be based on all possible response patterns, namely, the full contingency table. However, it is required that the expected frequency in each cell should be large, i.e., usually exceeding 5, for χ^2^ and *G*^2^ to be effective. This indicates that only a few items and a large number of examinees are required for a test using DCMs because even a small number of items can contribute to a large number of response patterns, a situation that easily leads to the sparseness of the contingency table. As such, some approaches have been proposed to address the issue of sparseness. For instance, although the Monte Carlo resampling technique can be used to produce empirical *p*-values (Tollenaar and Mooijaart, 2003), it is too time-consuming in practice. Another approach, the posterior predictive model checking method (Sinharay and Almond, 2007), is conservative and requires intensive computations due to the Markov chain Monte Carlo (MCMC) algorithm.

The limited-information tests are promising for model-data fit testing in DCMs. Unlike the full-information statistics, such as χ^2^ and *G*^2^, which use the entire contingency table, limited-information statistics use only some subset of lower-order marginal tables. *M*_*2*_ is a commonly used limited-information statistic that demonstrates good performance for model-data fit tests (Maydeu-Olivares and Joe, 2005; Cai et al., 2006). Specifically, because *M*_*2*_ uses only the univariate and bivariate marginal information, it can be better calibrated for the sparseness in the contingency table (Maydeu-Olivares and Joe, 2006). The performance of *M*_*2*_ has been sufficiently investigated under the structural equation model (SEM) and item response theory (IRT) framework in previous studies (e.g., Maydeu-Olivares and Joe, 2005; Maydeu-Olivares et al., 2011). However, the application of *M*_*2*_ in DCMs has emerged only in recent years (e.g., Jurich, 2014; Hansen et al., 2016; Liu et al., 2016), through which the usefulness of *M*_*2*_ in DCMs has been validated.

*M*_*2*_ is the most popular statistic of one family of limited-information test statistics, *M*_*r*_, where r denotes the marginal order (Maydeu-Olivares and Joe, 2006). Even though *M*_*2*_ uses only the univariate and bivariate marginal information, it is adequately powerful and can be computed efficiently (Maydeu-Olivares and Joe, 2005). Similar to traditional full-information statistics, the limited-information statistics are constructed using the residuals between observed and expected marginal probabilities. Hence, the residuals of the univariate and bivariate marginal probabilities should calculate first prior to the computation of *M*_*2*_. Let ${\dot{\pi }}_{1}=({\dot{\pi }}_{1},\ldots ,{\dot{\pi }}_{j},\ldots ,{\dot{\pi }}_{J}{)}^{\prime }$ denotes the first-order marginal probabilities, specifically, the marginal probabilities of correctly responding to each single test item, where ${\dot{\pi }}_{J}$ denotes the marginal probability of correctly responding to the *j*th item. Accordingly, ${\dot{\pi }}_{2}$ denotes the second-order marginal probabilities of correctly responding to each item pair. Then, let π  2=(π˙  1′,π˙  2′)′ denote the up-to-order 2 joint marginal probabilities and π denote the true response pattern probabilities, which is a 2^*J*^ × 1 vector. Thus, the univariate and bivariate marginal probabilities can be calculated as follows: **π**_2_ = **L**_2_ × **π** where **L**_2_ is a *d* × 2^*J*^ operator matrix of 1 and 0 s. The row-dimension, *d* = *J* + *J*(*J* − 1)/2, is the number of first- and second-order residuals. Using a test of 3 items as an example, the up-to-order 2 marginal probabilities should be

(7) $$\pi_{2}=\left(\begin{array}{c} {\dot{\pi }}_{1}\\ {\dot{\pi }}_{2} \end{array}\right)=\left(\begin{array}{c} {\dot{\pi }}_{1}\\ {\dot{\pi }}_{2}\\ {\dot{\pi }}_{3}\\ {\dot{\pi }}_{1,2}\\ {\dot{\pi }}_{1,3}\\ {\dot{\pi }}_{2,3} \end{array}\right)=L_{2}\times \pi =\left(\begin{array}{cccccccc} 0 & 1 & 0 & 0 & 1 & 1 & 0 & 1\\ 0 & 0 & 1 & 0 & 1 & 0 & 1 & 1\\ 0 & 0 & 0 & 1 & 0 & 1 & 1 & 1\\ 0 & 0 & 0 & 0 & 1 & 0 & 0 & 1\\ 0 & 0 & 0 & 0 & 0 & 1 & 0 & 1\\ 0 & 0 & 0 & 0 & 0 & 0 & 1 & 1 \end{array}\right)\;\left(\begin{array}{c} \pi_{\left(0,0,0\right)}\\ \pi_{\left(1,0,0\right)}\\ \pi_{\left(0,1,0\right)}\\ \pi_{\left(0,0,1\right)}\\ \pi_{\left(1,1,0\right)}\\ \pi_{\left(1,0,1\right)}\\ \pi_{\left(0,1,1\right)} \end{array}\right),$$

where π _(*#, #, #*)_ refers to the probability of the corresponding item response pattern.

Then, let **p**_2_ = **L**_2_ × **p** and ${\hat{\pi }}_{2}={\text{L}}_{2}\times \pi \left(\hat{\gamma }\right)$ indicate the observed and model-predicted marginal probabilities, respectively, where **p** refers to the observed response pattern probabilities and $\pi \left(\hat{\gamma }\;\right)$ refers to the model-predicted response pattern probabilities evaluated using the maximum likelihood estimate $\hat{\gamma }\;$. Thus, the univariate and bivariate residuals are computed as ${\text{r}}_{2}={\text{p}}_{2}-{\hat{\pi }}_{2}$. Thereafter, the *M*_*2*_ statistic is derived using **r**_2_ and a weight matrix, **W**_2_, as follows:

(8) $$M_{2}=N{\left(r_{2}\right)}^{\prime }W_{2}(r_{2})$$

where ${\text{W}}_{2}=\Xi_{2}^{-1}-\Xi_{2}^{-1}\Delta_{2}{\left({\Delta_{2}}^{\prime }\Xi_{2}^{-1}\Delta_{2}\right)}^{-1}{\Delta_{2}}^{\prime }\Xi_{2}^{-1}$. The vector of the first- and second-order residuals *r*_2_ is asymptotically normally distributed with means of zero and a covariance matrix $\Xi_{2}-\Delta_{2}I^{-1}{\Delta {\;}^{\prime }}_{2}$ (Reiser, 1996; Maydeu-Olivares and Joe, 2005):

(9) $$\sqrt{N}(r_{2})\to N(0,\Xi_{2}-\Delta_{2}I^{-1}\Delta {\;}^{\prime }{}_{2})$$

where $\Xi_{2}={\text{L}}_{2}\Xi {{\text{L}}^{\prime }}_{2}$ and $\Xi =\mathrm{diag}\left[\pi \left(\hat{\gamma }\;\right)\right]-\pi \left(\hat{\gamma }\;\right)\pi {\left(\hat{\gamma }\;\right)}^{\prime }$. Another component in **W**_2_, **Δ**_2_, is the first-order partial derivative of the expected marginal probabilities with respect to the maximum likelihood estimate of item parameters:

(10) $$\Delta_{2}=L_{2}\frac{\partial \pi \left(\hat{\gamma }\right)}{\partial \hat{\gamma }\;}.$$

The statistic is asymptotically distributed chi-squared with *d*−*k* degrees of freedom (Maydeu-Olivares and Joe, 2005), where *k* refers to the number of free parameters in the model. For a more elaborate description of *M*_*2*_, please refer to Hansen et al. (2016).

Similar to the traditional fit test statistics, the RMSEA_*2*_ can be calculated for *M*_*2*_ (Maydeu-Olivares and Joe, 2014). RMSEA_*2*_ is recommended to assess the approximate goodness-of-fit when *M*_*2*_ indicates the model does not fit exactly in the population. RMSEA_*2*_ can be obtained based on the observed ${\hat{M}}_{2}$ and the *df* :

(11) $${\text{RMSEA}}_{2}=\sqrt{\text{Max}\left(\frac{{\hat{M}}_{2}-df}{N\times df},0\right)}.$$

In this study, the information matrix, I, is the expected (Fisher) information matrix, which is described in a recent study in detail (Liu et al., 2018).

## Simulation study 1: the type I error rates of *M*_*2*_

Simulation 1 was conducted to examine the empirical Type I error rates of *M*_*2*_ when models were correctly specified under the condition of different attribute hierarchies and sample sizes. For each simulation, three sample sizes, *N* = 1,000, *N* = 2,000 and *N* = 4,000, and a fixed test length, *J* = 20, were considered with 500 replications. All simulations were performed by R and Mplus.

In this simulation, data were generated from five models: linear, divergent, convergent and unstructured HDCMs, and LCDM (no attribute hierarchy). The Q-matrix (see Table 1) used in this study involved 5 attributes and 20 items with each item measuring one, two or three attributes. In the Q-matrix, the number of corresponding items for each attribute was specified to be equal. In addition, the attributes were specified to follow a multidimensional normal distribution, with the mean vectors randomly selected from the uniform distribution μ (−.5,.5). We used 0 as the critical value to dichotomize the attribute vectors. With respect to the attribute correlations, as suggested by Kunina-Habenicht et al. (2012), 0.5 and 0.8 are typical low and high attribute correlation coefficients, respectively; therefore, the correlation coefficients between the attributes were randomly selected from μ(.5,.8)for each replication. To avoid the effects of the magnitudes of item parameters on the simulation results, the values of all main effects for each item were fixed at a value of 2, the values of all interaction effects were fixed at a value of 1, and the intercepts were fixed at a value of −0.5 times the sum of all main and interaction effects for each item (Templin and Bradshaw, 2014).

**Table 1:** Q-matrix with 5 attributes and 20 items.

**Item**	**V1**	**V2**	**V3**	**V4**	**V5**
1	1	0	0	0	0
2	0	1	0	0	0
3	0	0	1	0	0
4	0	0	0	1	0
5	0	0	0	0	1
6	1	1	0	0	0
7	0	0	0	1	1
8	0	1	1	0	0
9	0	0	1	1	0
10	0	0	0	1	1
11	1	1	1	0	0
12	1	1	0	0	1
13	1	0	0	1	1
14	1	0	1	1	0
15	0	0	1	0	1
16	1	0	1	0	0
17	0	1	0	1	0
18	0	1	0	1	0
19	0	1	0	0	1
20	1	0	1	0	1

Table 2 presents the results of the Type I error rates of *M*_*2*_ under the conditions of different attribute hierarchy types at five significance levels. The Type I error rates of *M*_*2*_ matched their expected rates well under different combinations of simulation conditions. Specifically, there was no substantial discrepancy in the performance of the Type I error rate control of *M*_*2*_ for the five different attribute hierarchy types. The average values of the empirical Type I error rates at the five significance levels were 0.014, 0.057, 0.111, 0.220, and 0.271, respectively. Hence, it is evident that the *M*_*2*_ statistic exhibited good Type I error rate control for different attribute hierarchy types. However, further examination of the empirical Type I error rates found that *M*_*2*_ under the LCDM, which indicates the independent relationships for attributes, had slightly better Type I error rate control compared to HDCMs.

**Table 2:** Type I error rates for HDCMs with five different hierarchical structures.

**Hierarchy type**	***N***	***df***	**Mean**	***SD***	**Empirical rejection rate**				
					**0.010**	**0.050**	**0.100**	**0.200**	**0.250**
Linear	1,000	145	146.50	17.75	0.020	0.068	0.120	0.234	0.294
	2,000	145	144.81	17.22	0.014	0.042	0.086	0.182	0.246
	4,000	145	145.27	16.43	0.002	0.040	0.100	0.206	0.256
Divergent	1,000	133	135.64	16.37	0.016	0.060	0.118	0.238	0.298
	2,000	133	135.31	16.97	0.018	0.074	0.140	0.252	0.292
	4,000	133	135.25	17.47	0.018	0.074	0.136	0.260	0.308
Convergent	1,000	142	142.36	18.21	0.022	0.068	0.120	0.230	0.264
	2,000	142	142.24	17.29	0.012	0.052	0.108	0.222	0.264
	4,000	142	142.28	17.30	0.008	0.046	0.100	0.214	0.264
Unstructured	1,000	121	122.05	16.06	0.020	0.064	0.114	0.216	0.278
	2,000	121	122.09	15.96	0.018	0.064	0.114	0.222	0.272
	4,000	121	121.62	15.78	0.008	0.068	0.108	0.212	0.276
No Hierarchy	1,000	89	89.44	13.20	0.012	0.050	0.106	0.212	0.260
	2,000	89	88.59	13.19	0.008	0.032	0.094	0.202	0.262
	4,000	89	89.35	12.57	0.012	0.046	0.094	0.200	0.234

*N, the sample size; df, the degrees of freedom; SD, Standard Deviation*.

## Simulation study 2: the statistical power of *M*_*2*_

Simulation 2 was conducted to examine the power of *M*_*2*_ under the conditions of model and Q-matrix misspecifications. The model and Q-matrix misspecifications were regarded as the sources of model–data misfit for DCMs in previous studies (e.g., Kunina-Habenicht et al., 2012; de la Torre and Lee, 2013). Especially, the Q-matrix misspecification can be a very influential source of model-data misfit (Kunina-Habenicht et al., 2012).

The generating models and the Q-matrix in this simulation were identical to those in Simulation 1 except that the LCDM was no longer considered. Identical to Simulation 1, three sample sizes, *N* = 1,000, *N* = 2,000, and *N* = 4,000, and a fixed test length, *J* = 20, were considered, and each simulation was performed with 500 replications. In addition, according to previous findings (e.g., Kunina-Habenicht et al., 2012; Liu et al., 2016), attribute correlations may affect the power of model-data fit statistics in DCMs, we therefore considered three attribute correlation levels, 0.3, 0.5, and 0.8, to examine the effects of different attribute correlations on the power of *M*_*2*_.

Regarding the model misspecification, we used the DINA model as the misspecified fitting model. The DINA model classifies the examinees into two groups: the examinees who have mastered all measured attributes and those who have not mastered at least one of the measured attributes. With respect to the Q-matrix misspecification, we used the random balance design (Chen et al., 2013) to misspecify 20% of the Q-matrix elements. This means that some attributes that were originally measured by the items are no longer required in the misspecified Q-matrix, and vice versa (see Table 3). The other technical settings are presented in the previous simulation study.

**Table 3:** Random balance design of Q-matrix misspecification.

***K*_*j*_**	**Alterations**	**Note**
1	*q*_*jk*_ = 0 → *q*_*jk*_ = 1	Over-specification
2	*q*_*jk*_ = 1 → *q*_*jk*_ = 0	Under-specification
3	*q*_*jk*_ = 0 → *q*_*jk*_ = 1, $q_{jk^{\prime }}=1\to q_{jk^{{}^{\prime }}}=0$	Both

*K_j_ is the number of required attributes for the j th item; q_jk_ is the entry in the jth row and kth column of the Q-matrix; $q_{jk^{{}^{\prime }}}$is the entry in the jth row and k'th column of the Q-matrix, k ≠ k′[this table is excerpted from and with the permission of Liu et al. (2016)]*.

Table 4 presents the results of the empirical rejection rates of *M*_*2*_ when the DINA model is used as the misspecified model. According to the table, for each attribute hierarchy, the statistical power increased with the increase of the sample size. This trend existed across different attribute correlations and significance levels. Specifically, when the sample size was 4,000, *M*_*2*_ had good performance in detecting the misspecified model. However, when the sample sizes were 2,000 and 1,000, the statistical power of *M*_*2*_ was unsatisfactory. In addition, the statistical power of *M*_*2*_ for the unstructured HDCM was rather poor in this simulation. Table 5 presents the results under the Q-matrix misspecification. According to Table 5, it is noted that the statistical power of *M*_*2*_ was extremely high for each type of attribute hierarchy. Specifically, the power for the divergent and unstructured HDCMs reached 100%, and the power for the linear and convergent HDCMs was slightly < 100% only when the sample size was 1,000. Generally, the attribute correlations had no effects on the statistical power of *M*_*2*_ for each type of misfit.

**Table 4:** The empirical rejection rates of *M*_*2*_ when DINA as the misspecified model.

**Hierarchy type**	***r***	***N***	***df***	**Mean**	***SD***	**Empirical rejection rate**				
						**0.010**	**0.050**	**0.100**	**0.200**	**0.250**
Linear	0.3	1,000	139	145.38	17.96	0.036	0.108	0.192	0.318	0.398
		2,000	139	151.38	18.91	0.058	0.184	0.288	0.468	0.530
		4,000	139	168.80	22.82	0.290	0.522	0.626	0.758	0.800
	0.5	1,000	139	144.63	17.98	0.036	0.110	0.182	0.328	0.368
		2,000	139	151.84	18.79	0.076	0.198	0.306	0.468	0.532
		4,000	139	168.28	22.77	0.264	0.490	0.626	0.756	0.804
	0.8	1,000	139	144.32	17.20	0.024	0.088	0.166	0.308	0.360
		2,000	139	149.42	19.90	0.072	0.172	0.266	0.418	0.480
		4,000	139	167.11	23.32	0.266	0.476	0.586	0.710	0.748
Divergent	0.3	1,000	139	149.67	17.75	0.050	0.154	0.250	0.412	0.480
		2,000	139	166.32	21.12	0.220	0.466	0.604	0.732	0.776
		4,000	139	197.93	23.20	0.778	0.906	0.958	0.986	0.992
	0.5	1,000	139	149.22	17.84	0.040	0.156	0.244	0.420	0.472
		2,000	139	167.34	20.60	0.238	0.490	0.598	0.746	0.786
		4,000	139	197.40	24.08	0.746	0.892	0.946	0.968	0.980
	0.8	1,000	139	151.04	17.88	0.048	0.204	0.294	0.450	0.498
		2,000	139	166.49	21.36	0.246	0.464	0.584	0.730	0.784
		4,000	139	198.51	22.56	0.788	0.932	0.958	0.982	0.984
Convergent	0.3	1,000	139	189.44	19.51	0.144	0.320	0.466	0.608	0.676
		2,000	139	180.96	23.07	0.486	0.696	0.798	0.890	0.912
		4,000	139	229.92	28.10	0.968	0.991	0.996	0.998	0.998
	0.5	1,000	139	157.22	20.34	0.128	0.280	0.396	0.564	0.628
		2,000	139	182.73	23.27	0.526	0.738	0.832	0.898	0.924
		4,000	139	227.27	28.05	0.962	0.992	1.00	1.00	1.00
	0.8	1,000	139	159.87	21.02	0.152	0.342	0.464	0.606	0.664
		2,000	139	182.15	21.81	0.530	0.748	0.838	0.906	0.924
		4,000	139	230.07	27.73	0.962	0.994	1.00	1.00	1.00
Unstructured	0.3	1,000	139	133.57	16.32	0.006	0.018	0.052	0.110	0.166
		2,000	139	135.12	15.96	0.000	0.026	0.068	0.124	0.178
		4,000	139	141.53	16.91	0.012	0.064	0.130	0.248	0.304
	0.5	1,000	139	133.94	15.99	0.002	0.028	0.056	0.130	0.162
		2,000	139	136.63	16.26	0.000	0.036	0.088	0.180	0.216
		4,000	139	139.11	16.96	0.014	0.050	0.100	0.202	0.256
	0.8	1,000	139	133.75	16.60	0.004	0.030	0.056	0.116	0.150
		2,000	139	135.51	15.33	0.004	0.030	0.056	0.138	0.184
		4,000	139	139.59	16.68	0.008	0.060	0.112	0.212	0.256

*r refers to the attribute correlations*.

**Table 5:** The empirical rejection rates of *M*_*2*_ for the Q-matrix misspecification.

**Hierarchy type**	***r***	***N***	***df***	**Mean**	***SD***	**Empirical rejection rate**				
						**0.010**	**0.050**	**0.100**	**0.200**	**0.250**
Linear	0.3	1,000	145	202.01	23.83	0.722	0.884	0.938	0.968	0.976
		2,000	145	262.38	28.27	1.00	1.00	1.00	1.00	1.00
		4,000	145	384.27	37.32	1.00	1.00	1.00	1.00	1.00
	0.5	1,000	145	205.84	22.33	0.780	0.930	0.972	0.990	0.994
		2,000	145	261.91	28.80	0.998	1.00	1.00	1.00	1.00
		4,000	145	380.55	36.98	1.00	1.00	1.00	1.00	1.00
	0.8	1,000	145	204.77	24.25	0.758	0.902	0.950	0.972	0.984
		2,000	145	261.99	26.38	0.998	1.00	1.00	1.00	1.00
		4,000	145	379.51	34.44	1.00	1.00	1.00	1.00	1.00
Divergent	0.3	1,000	134	345.94	40.64	1.00	1.00	1.00	1.00	1.00
		2,000	134	561.30	54.83	1.00	1.00	1.00	1.00	1.00
		4,000	134	990.87	76.28	1.00	1.00	1.00	1.00	1.00
	0.5	1,000	134	348.45	38.43	1.00	1.00	1.00	1.00	1.00
		2,000	134	562.48	57.23	1.00	1.00	1.00	1.00	1.00
		4,000	134	995.40	73.00	1.00	1.00	1.00	1.00	1.00
	0.8	1,000	134	346.82	39.92	1.00	1.00	1.00	1.00	1.00
		2,000	134	561.81	56.06	1.00	1.00	1.00	1.00	1.00
		4,000	134	1001.17	76.38	1.00	1.00	1.00	1.00	1.00
Convergent	0.3	1,000	141	213.81	27.01	0.874	0.966	0.988	0.996	0.996
		2,000	141	283.18	32.61	1.00	1.00	1.00	1.00	1.00
		4,000	141	418.32	42.50	1.00	1.00	1.00	1.00	1.00
	0.5	1,000	141	212.96	26.70	0.876	0.978	0.996	1.00	1.00
		2,000	141	283.52	32.27	1.00	1.00	1.00	1.00	1.00
		4,000	141	427.45	42.58	1.00	1.00	1.00	1.00	1.00
	0.8	1,000	141	211.03	26.10	0.854	0.940	0.970	0.988	0.994
		2,000	141	283.28	32.45	1.00	1.00	1.00	1.00	1.00
		4,000	141	424.18	40.05	1.00	1.00	1.00	1.00	1.00
Unstructured	0.3	1,000	122	381.89	53.07	1.00	1.00	1.00	1.00	1.00
		2,000	122	635.09	83.75	1.00	1.00	1.00	1.00	1.00
		4,000	122	1157.21	125.80	1.00	1.00	1.00	1.00	1.00
	0.5	1,000	122	382.36	54.08	1.00	1.00	1.00	1.00	1.00
		2,000	122	639.16	81.81	1.00	1.00	1.00	1.00	1.00
		4,000	122	1167.02	133.01	1.00	1.00	1.00	1.00	1.00
	0.8	1,000	122	384.04	53.74	1.00	1.00	1.00	1.00	1.00
		2,000	122	638.19	82.66	1.00	1.00	1.00	1.00	1.00
		4,000	122	1161.45	136.10	1.00	1.00	1.00	1.00	1.00

*r refers to the attribute correlations*.

## Empirical illustration

We used the Examination for the Certificate of Proficiency in English (ECPE) data (Templin and Bradshaw, 2014) to investigate the usefulness of *M*_*2*_ in real settings. The ECPE data is publicly available in the *CDM* package in R (Robitzsch et al., 2014). The ECPE data embrace three attributes (knowledge of morphosyntactic rules, cohesive rules and lexical rules), 28 multiple-choice items and 2,922 examinees (Buck and Tatsuoka, 1998). The Q-matrix of the ECPE data is presented in Table 6. There exists a linear hierarchy underlying the three attributes: “lexical rules” is the prerequisite attribute of “cohesive rules,” which in turn is the prerequisite attribute of “morphosyntactic rules.” For illustration purposes, we used the linear HDCM, LCDM, DINA, and C-RUM to fit the data and applied *M*_*2*_ and RMSEA_*2*_ to evaluate the test-level model-data fit. As mentioned previously, The LCDM is the saturated model which involves the largest number of item parameters. The DINA model is a parsimonious model which includes two parameters for each item (the guessing and slip parameters). The C-RUM (Hartz, 2002) can be obtained from LCDM by retaining all main effects and removing all interaction effects of attributes. Modeling of LCDM, DINA, and C-RUM can be fulfilled by the *gdina* function in the *CDM* package in R. For the linear HDCM, because there is no available function in the *CDM* package, Mplus was used for the modeling according to the work by Templin and Hoffman (2013). We provided an abbreviated Mplus Syntax for the estimation of ECPE data and the R function for calculating *M*_*2*_ in the online Supplementary Material. The full Mplus Syntax can be accessed at the personal website of the developer of HDCM (https://jonathantemplin.com/hierarchical-attribute-structures/).

**Table 6:** Q-matrix of the ECPE data.

**Item**	**V1**	**V2**	**V3**	**Item**	**V1**	**V2**	**V3**
1	1	1	0	15	0	0	1
2	0	1	0	16	1	0	1
3	1	0	1	17	0	1	1
4	0	0	1	18	0	0	1
5	0	0	1	19	0	0	1
6	0	0	1	20	1	0	1
7	1	0	1	21	1	0	1
8	0	1	0	22	0	0	1
9	0	0	1	23	0	1	0
10	1	0	0	24	0	1	0
11	1	0	1	25	1	0	0
12	1	0	1	26	0	0	1
13	1	0	0	27	1	0	0
14	1	0	0	28	0	0	1

Table 7 presents the item parameter estimates of the ECPE data using the LCDM, DINA, and C-RUM models. In this study, we used the same estimation procedure programmed with Mplus as Templin and Bradshaw (2014) for the HDCM estimation. Thus, the item parameter estimates of the HDCM are available in Templin and Bradshaw (2014). The values of *M*_*2*_ and RMSEA_*2*_ for these models are presented in Table 8. Unfortunately, the *M*_*2*_ statistic rejected all models for the ECPE data. However, the values of RMSEA_*2*_ were small. In addition, the relative model-data fit statistics, AIC and BIC, showed that HDCM was the best fitting model for the ECPE data.

**Table 7:** Item parameters of the ECPE data by LCDM, DINA and C-RUM.

	**LCDM**							**DINA**		**C-RUM**			
***j***	**0**	**1(1)**	**1(2)**	**1(3)**	**2(12)**	**2(13)**	**2(23)**	***g***	***s***	**0**	**1(1)**	**1(2)**	**1(3)**
1	0.84	−1.39	0.56		2.76			0.71	0.21	0.69	0.11	0.12	
2	1.02		1.24					0.74	0.17	0.74		0.17	
3	−0.35	1.26		0.36		0.02		0.44	0.30	0.41	0.28		0.09
4	−0.14			1.68				0.48	0.36	0.46			0.36
5	1.08			2.01				0.76	0.20	0.75			0.21
6	0.86			1.69				0.72	0.22	0.70			0.22
7	−0.09	2.73		0.94		-0.82		0.54	0.37	0.49	0.24		0.22
8	1.46		1.89					0.81	0.15	0.81		0.15	
9	0.11			1.20				0.53	0.27	0.53			0.26
10	0.06	2.05						0.49	0.35	0.52	0.38		
11	−0.03	0.50		0.95		1.10		0.56	0.35	0.49	0.21		0.22
12	−1.74	−21.29		1.26		22.81		0.19	0.50	0.13	0.34		0.26
13	0.66	1.63						0.63	0.24	0.66	0.25		
14	0.18	1.37						0.52	0.27	0.55	0.28		
15	0.99			2.11				0.75	0.21	0.73	0.23		
16	−0.08	1.50		0.87		-0.01		0.55	0.33	0.49	0.22		0.20
17	1.32		1.42	0.62			-0.61	0.82	0.13	0.80		0.08	0.07
18	0.92			1.38				0.73	0.19	0.71			0.20
19	−0.20			1.85				0.47	0.38	0.45			0.39
20	−1.38	−0.09		0.90		1.73		0.24	0.47	0.19	0.38		0.19
21	0.17	1.09		1.13		-0.01		0.62	0.28	0.55	0.13		0.24
22	−0.88			2.24				0.32	0.49	0.30			0.50
23	0.65		2.01					0.66	0.27	0.66		0.28	
24	−0.67		1.48					0.33	0.36	0.33		037	
25	0.10	1.13						0.51	0.22	0.52		0.25	
26	0.16			1.11				0.55	0.23	0.54			0.24
27	−0.88	1.72						0.27	0.36	0.30	0.40		
28	0.56			1.74				0.66	0.26	0.64			0.27

*For the LCDM and C-RUM, 0 refers to the intercept, “1(#)” refers to the main effect, and “2(##)” refers to the two-way interaction effect; for the DINA model, “g” refers to the “guessing” parameter and “s” refers to the “slipping” parameter*.

**Table 8:** *M*_*2*_ and RMSEA_*2*_ statistics for the ECPE data.

**Model**	***M_***2***_***	***df***	***p***	**RMSEA_***2***_**	**90% CI**	**AIC**	**BIC**
HDCM	514.280	338	0.000	0.013	[0.011,0.016]	85,639	86,045
LCDM	470.809	325	0.000	0.012	[0.010,0.015]	85,639	86,124
DINA	515.607	343	0.000	0.013	[0.011,0.015]	85,809	86,186
C-RUM	504.859	334	0.000	0.013	[0.011,0.016]	85,634	86,064

## Discussion

This article aims to investigate the performance of a widely used limited-information fit test statistic, *M*_*2*_, in the hierarchical DCMs. We used the HDCMs to model the four fundamental attribute hierarchies and conducted two simulation studies and one empirical study to testify to the usefulness of *M*_*2*_.

According to Simulation 1, the observed Type I error rates of *M*_*2*_ are reasonably close to the nominal levels for each attribute hierarchy. This indicates that the *M*_*2*_ statistic can be safely used for different types of attribute hierarchies in DCMs. The attribute hierarchies are of great importance for practitioners using DCMs because hierarchical structures often exist among different knowledge, skills and psychological concepts. However, researchers and practitioners often improperly assume that the attributes involved in the cognitive diagnostic tests are independent. Hence, by examining four fundamental types of attribute hierarchies, in an initial step, we demonstrated the usefulness of *M*_*2*_ for addressing the complex attribute relationships in DCMs. Our findings echo previous findings on Type I error rates of *M*_*2*_ in DCMs (e.g., Hansen et al., 2016; Liu et al., 2016). In the study by Hansen et al. (2016), the Type I error rates of *M*_*2*_ for higher-order DINA, DINA and their variations were close to what would be expected. It should be noted that the Type I error rates of *M*_*2*_ were examined for a fixed test length, 20 items, which is close to that of the study by Hansen et al. (2016), 24 items. The test length was decided to cause the sparseness of the contingency table, based on which the limited-information statistics can be used. However, readers who are interested in how test length affects the Type I error rates of *M*_*2*_ in DCMs, should refer to the study by Liu et al. (2016). In their study, given both non-sparse (*J* = 6) and sparse contingency tables (*J* = 30/50), *M*_*2*_ demonstrated good control of Type I error rates.

Thereafter, we further examined the sensitivity of *M*_*2*_ to the specification of an incorrect DINA model and the misspecification of the Q-matrix. According to Simulation 2, the *M*_*2*_ statistic is extremely sensitive to the misspecification of the Q-matrix regardless of sample size and attribute correlation. This finding is consistent with previous studies (Hansen et al., 2016; Liu et al., 2016) that emphasize the importance of the correct specification of the Q-matrix in cognitive diagnostic tests. It should be noted that our study adopted the same approach for the Q-matrix misspecification generation as used by previous studies (e.g., Chen et al., 2013; Liu et al., 2016). The percentage of misspecified Q-matrix elements were set to be 20% considering that the true Q-matrix may not be easily identified by domain experts in reality. Thus 20% of misspecified Q-matrix elements was designed to reflect a substantial Q-matrix misspecification. In addition, due to the fact that the parameter estimation of a single HDCM by Mplus is extremely time-consuming (20–60 min), we did not examine the power of *M*_*2*_ for other levels of misspecified Q-matrix elements considering the infeasible simulation time. However, the study by Hansen et al. (2016) provided the evidence that when only two elements of Q-matrix were misspecified, the empirical rejection rates of *M*_*2*_ reached 100% in most simulation conditions, whereas omitting an existing attribute or adding an extraneous attribute would lead to lower sensitivity of *M*_*2*_ to Q-matrix specification. This finding implies that *M*_*2*_ may be largely sensitive to the Q-matrix misspecification given any percentage of misspecified Q-matrix elements. Future studies are encouraged to investigate how different types and different levels of Q-matrix misspecification affect the statistical power of *M*_*2*_ in DCMs.

With respect to model misspecification, when the DINA model was used as the fitting model, *M*_*2*_ was sensitive to the misspecification for large sample sizes. This expected finding can be explained by different assumptions regarding the relationships between the items and attributes underlying the DINA and HDCMs. Specifically, the DINA model is a non-compensatory model, which indicates that an examinee must possess mastery of all required attributes to correctly respond to some items and that the lack of any one of the required attributes will contribute to an incorrect answer. In contrast, the relationships between items and attributes of the HDCMs are compensatory, indicating that examinees have a higher probability of correctly responding to an item when they have mastered any one of the additional required attributes of the item. Accordingly, *M*_*2*_ was generally sensitive to the specification of the incorrect DINA model due to the huge discrepancy regarding the natures of the DINA and HDCMs. In addition, it is evident that larger sample sizes generally lead to higher statistical power, a finding that is consistent with previous studies (Kunina-Habenicht et al., 2012; Liu et al., 2016). Furthermore, we found that the attribute correlations have no noticeable influence on the sensitivity of *M*_*2*_ to the model and Q-matrix misspecifications. It is possible that the attribute hierarchies already assume strong relationships among the attributes and therefore the specified attribute correlation levels do not affect the performance of *M*_*2*_. This finding echoes previous findings that attribute correlations would not significantly influence the classification accuracy of DCMs (Kunina-Habenicht et al., 2012) and the statistical power of *M*_*2*_ in DCMs (Liu et al., 2016). It should be noted that in the study by Liu et al. (2016), when the sample size was small, a lower attribute correlation would lead to slightly higher power of *M*_*2*_. However, the opposite result was observed for a small sample size in our study. This is possibly due to that small sample sizes would lead to unstable parameter estimation which in in turn affected the classification accuracy and the performance of *M*_*2*_.

Regarding the empirical illustration, the *M*_*2*_ statistic rejected all models. It was expected that *M*_*2*_ would reject models except HDCM because the ECPE data involves hierarchical attribute relationships. One possible explanation is that *M*_*2*_ is, in practice, a strongly sensitive fit test statistic. Undoubtedly, the true generating Q-matrix for the ECPE data cannot be known. Hence, it is unavoidable that there exist some mistakes in the Q-matrix, which is specified by the domain and measurement experts. Moreover, the simulation study shows that *M*_*2*_ is extremely sensitive to the Q-matrix misspecification. So it is not surprising that *M*_*2*_ rejected all models for the ECPE data. The evidence of this finding is also supported by the empirical illustrations of numerous studies (e.g., Cai et al., 2006; Maydeu-Olivares et al., 2011; Jurich, 2014). Considering the sensitivity of *M*_*2*_, which provides only information about whether the models fit the data, many researchers recommended the use of RMSEA_*2*_ to assess the goodness of approximation of DCMs and to characterize the degree of model error (e.g., Maydeu-Olivares and Joe, 2014). The RMSEA_*2*_ is an effect-size-like index that can be used for the direct comparisons among the different models. According to Liu et al. (2016)'s criteria, the values of .030 and .045 are the thresholds for excellence and good fit, respectively. The values of RMSEA_*2*_ for the ECPE data in this study are significantly < 0.030, indicating good model-data fit for all models. However, it was expected that values of RMSEA_*2*_ would vary across the four models because they assume different relationships between attributes and items. For example, LCDM is the general modeling framework whereas DINA is one of the most parsimonious models which defines only two item parameters. Despite the fact that RMSEA penalizes the model complexity and measures the degree of model-data misfit, it was found to be influenced by sample sizes and the degree of freedom (*df*) in other modeling frameworks (e.g., structural equation modeling). For small sample sizes and small *df*, the RMSEA is often positively biased (Kenny et al., 2015). However, it was also evident that compared with small sample sizes and small *df*, the model rejection rates of RMSEA were much lower for large sample sizes and large *df* given the same cutoff value (Chen et al., 2008). Therefore, in our study, it is possible that the large sample size and the large *df* s led to indistinguishable values and CIs of RMSEA_*2*_. More empirical investigations are needed for revealing the performance of RMSEA_*2*_ in examining the model-data fit for DCMs.

For the real-life application of DCMs, practitioners should carefully examine the relationships between attributes at the test design stage or the initial stage of data analysis. HDCMs are recommended as the modeling framework if attribute hierarchies are identified. However, the model selection decision should be based on both substantive considerations and technical solutions. Despite the fact that the model-data fit tests in DCMs are under-developed, according to our findings, the *M*_*2*_ statistic is of great value for examining the absolute model-data fit of HDCMs. However, given the strong sensitivity of *M*_*2*_ to model or Q-matrix errors, RMSEA_*2*_ is recommended to evaluate the model-data misfit.

Some limitations exist in the present study. First, we used a fixed test length of 20 items, which, in reality, is considered as a reasonable test length. However, future studies are encouraged to investigate the effects of test length on the performance of *M*_*2*_ in DCMs. Second, we considered only the dichotomous data in this study. Future studies regarding the application of *M*_*2*_ should include polytomous models. Finally, although four fundamental afttribute hierarchies were considered in our research, it is recommended that the performances of *M*_*2*_ and RMSEA_*2*_ be examined in DCMs with more complicated attribute relationships.

## Author contributions

FC and TX contributed to the conceptualization and design of the work. FC and YL contributed to the analysis and interpretation of data. FC, YL, YC, and TX were involved in drafting and revising the manuscript. All authors approved the final manuscript submitted.

### Conflict of interest statement

The authors declare that the research was conducted in the absence of any commercial or financial relationships that could be construed as a potential conflict of interest.
